# Clinical exome sequencing efficacy and phenotypic expansions involving non-isolated congenital anomalies of kidney and urinary tract (CAKUT+)

**DOI:** 10.1038/s41431-025-01929-3

**Published:** 2025-09-06

**Authors:** E. Andres Rivera-Munoz, Xiaonan E. Zhao, Jill A. Rosenfeld, Pamela N. Luna, Chad A. Shaw, Jennifer E. Posey, Daryl A. Scott

**Affiliations:** 1 Department of Molecular and Human Genetics, Baylor College of Medicine, Houston, TX, USA.; 2 Genetics and Genomics Graduate Program, Baylor College of Medicine, Houston, TX, USA.; 3 Baylor Genetics, Houston, TX, USA.

## Abstract

Congenital Anomalies of Kidney and Urinary Tract (CAKUT) can occur in isolation or in conjunction with one or more non-CAKUT associated congenital anomalies or neurodevelopmental disorders (CAKUT+). A molecular cause is not identified in most individuals with CAKUT+. This is due, in part, to uncertainty regarding the efficacy of genetic testing and an incomplete understanding of the genes that cause CAKUT+. Here, we use data from 515 individuals with CAKUT+ (*n* = 500) or isolated CAKUT (*n* = 15) to determine the efficacy of clinical exome sequencing (cES) and to identify new phenotype expansions that involve CAKUT. We determined that cES established a molecular diagnosis in 27.4% (141/515) of individuals in this cohort. No statistically significant difference in efficacy was seen with regards to age, sex, CAKUT phenotype, or associated organ system abnormality. Only 3.5% (5/144) to 14.6% (21/144) of the individual diagnoses made in our cohort could have been identified using one of four clinically available CAKUT gene panels. We then used a machine-learning approach to confirm that *PHIP* is a CAKUT gene and to implicate *ADNP* and *SETD5* as genes associated with an increased risk of CAKUT. These findings lead us to conclude that cES should be considered in individuals with CAKUT+ for whom a molecular diagnosis has not been identified, that cES has the potential to identify many diagnoses in individuals with CAKUT+ that would be missed using a CAKUT gene panel, and that individuals with *ADNP*-, *PHIP*-, and *SETD5*-related disorders may present with CAKUT phenotypes.

## INTRODUCTION

Congenital Anomalies of Kidney and Urinary Tract (CAKUT) affect 4–100 per 10,000 newborns and account for 20–30% of pediatric malformations [1–3]. The spectrum of CAKUT phenotypes is highly heterogenous [4–7]. While CAKUT may manifest in isolation, it frequently is accompanied by one or more non-CAKUT associated congenital anomalies or neurodevelopmental disorders (CAKUT+) [8–10].

CAKUT remains a leading cause of kidney failure in children and young adults [6, 8]. Undiagnosed CAKUT in children and young adults also plays a role in susceptibility to renal disease later in life [8, 11–13]. Identifying a molecular etiology for CAKUT+ in an individual has the potential to change medical management and is a prerequisite for accurate genetic counseling and prognostication [8, 14–16].

Over 100 genes have been implicated in monogenic forms of CAKUT and CAKUT+, and clinical ES (cES) has become a commonly ordered genetic test for individuals with a wide range of medical conditions [5, 9, 13, 15, 17, 18]. However, cES is not universally ordered for individuals with CAKUT+ who do not have a molecular diagnosis. Limited information regarding the diagnostic efficacy of cES in CAKUT+, and an incomplete understanding of the genes that contribute to CAKUT+ phenotypes, has likely limited the use of cES among these patients.

In the present study, we sought to address these deficiencies by evaluating the diagnostic efficacy of cES in individuals with CAKUT and CAKUT+, determining if a similar efficacy could have been achieved using commercially available CAKUT gene panels, and identifying novel phenotypic expansion involving CAKUT using clinical and molecular data from a large clinical cohort.

## SUBJECTS AND METHODS

### Cohort Identification and diagnostic review

We identified 515 individuals (CAKUT_P1-P515) with isolated CAKUT (*n* = 15) or CAKUT+ (*n* = 500) referred to Baylor Genetics for cES (Table S1). Clinical phenotypes were annotated with Human Phenotype Ontology (HPO) terms [19–22] and manually reviewed and grouped into seven categories: renal agenesis, renal duplication, lower urinary tract obstruction or anomalies, obstructive ureteral phenotypes, renal ectopy/malrotation/fusion, vesicoureteral reflux, multicystic dysplastic kidney, or multiple categories [2, 13].

Each case was reviewed to establish a level of confidence in the molecular diagnosis (definitive, probable, or provisional) based on the assessed pathogenicity of the molecular findings in the genetics report, expected mode of inheritance, and available phenotypic information as previously described (Table S1, Supplemental Materials) [23].

### Generating CAKUT-specific rank annotation scores

We employed a previously published machine learning algorithm to generate CAKUT-specific rank annotation scores [24]. This tool integrates annotations from various genome-scale knowledge sources to construct a pattern in genomic feature space based on a set of phenotype-associated training genes, then ranks all RefSeq genes with respect to their similarity to that pattern using quantitative similarity metrics [25–31]. Based on each gene’s annotation similarity, the algorithm generates a gene-, phenotype-specific rank annotation score between 0 and 100%.

**Fig. 1:**
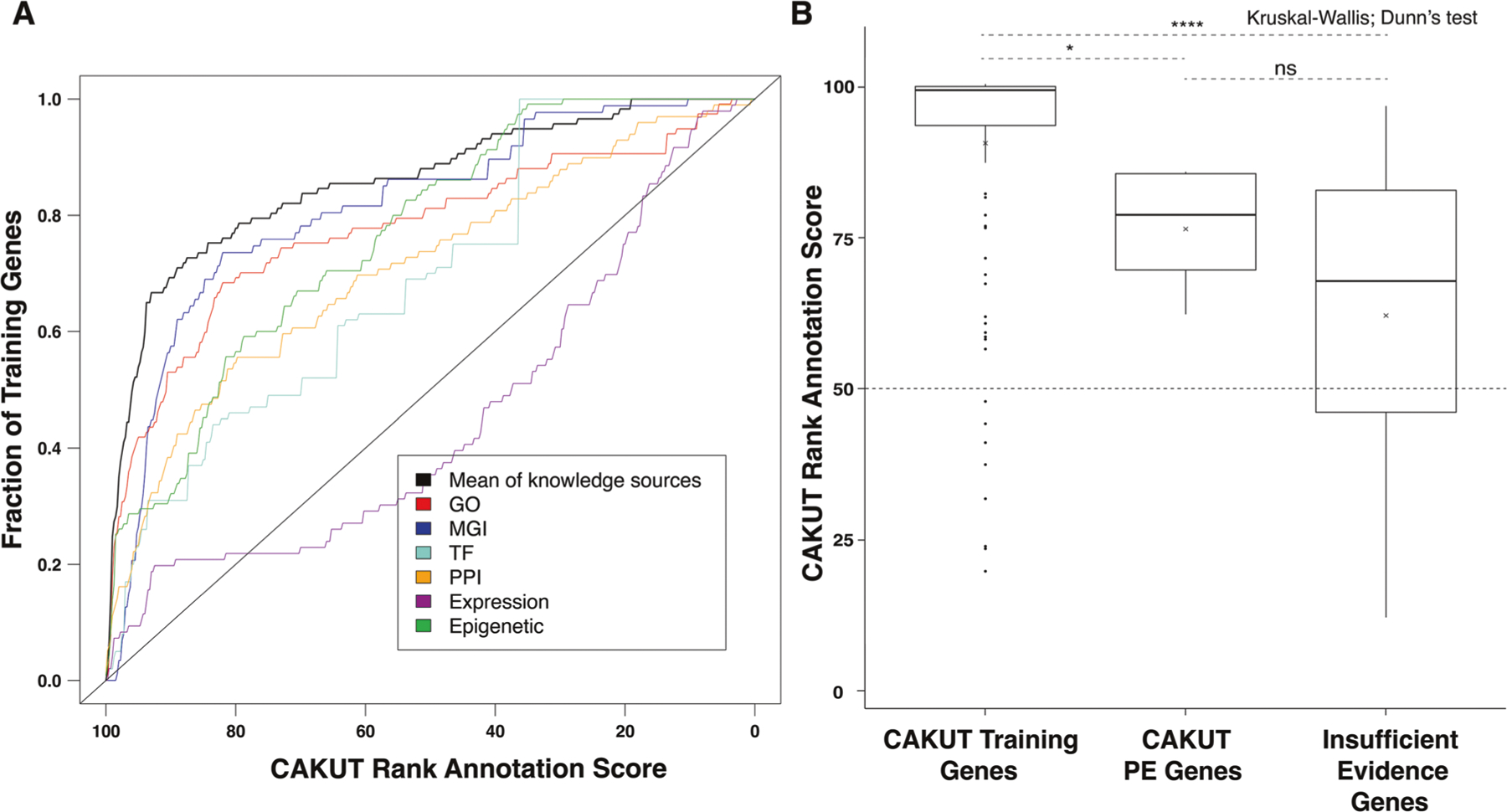
Ranking all RefSeq genes based on their similarity to a set of known CAKUT genes. A set of 117 genes clearly associated with CAKUT (Table S2) was used to train a previously published machine learning algorithm that integrates annotation data from various genome-scale knowledge sources [24]. **A** A leave-one-out validation was performed, and the resulting receiver operating characteristic (ROC) style curves were generated for each knowledgebase (colored lines) and an omnibus curve (black line) was generated using fit data from all knowledge sources. The area under each curve demonstrates performance above chance (black diagonal line). **B** Box plots comparing the CAKUT-specific rank annotation scores of genes in the training set, 4 high priority/phenotype expansion (PE) CAKUT candidate genes, and 55 other CAKUT candidate genes with insufficient evidence to suggest a phenotype expansion. Comparisons between groups were performed using a Wilcoxon rank-sum test to determine whether the distribution of scores significantly differed between these groups. GO Gene Ontology, MGI Mouse Genome Informatics, TF Transcription factor, PPI Protein-protein interaction. NS *p* > 0.05, * *p* ≤ 0.05, ** *p* ≤ 0.01, *** *p* ≤ 0.001, **** *p* ≤ 0.0001.

A curated list of 117 known CAKUT genes from the literature and commercial diagnostic CAKUT panels (Table S2) was used to train the algorithm. The resulting omnibus curve, produced using fit data from all knowledge sources, validates that the algorithm distinguishes between the CAKUT-associated genes in the training set and all other RefSeq genes better than random chance (Fig. 1A).

We generated CAKUT-specific rank annotation scores for all RefSeq genes (Table S3). The rank annotation scores of the training set genes ranged from 20% to 100% with a median score of 99% (Fig. 1B), compared to a median score of 50% across all RefSeq genes, demonstrating again, that the algorithm can distinguish between the 117 CAKUT-associated genes and all other RefSeq genes.

For further details, please see the Supplemental Subjects and Materials.

## RESULTS

### Efficacy of cES in individuals with CAKUT phenotypes

We identified 515 individuals with CAKUT who were referred to a clinical diagnostic laboratory for cES (Table S1). Most (500/515; 97.1%) had one or more non-CAKUT associated congenital anomalies or neurodevelopmental disorders and were designated as CAKUT+ (Table 1). These 515 individuals were divided into those with variants in genes putatively relevant to their phenotypes which were reported back to physicians (positive, *n* = 203), and those without putatively relevant variants identified (negative, *n* = 312) (Fig. S1). The distributions of age at genetic testing and sex between positive and negative cases were not significantly different (*p* value = 0.9004, T-test, and *p* value = 0.4075, Chi-square test, respectively).

In our cohort, 27.4% (141/515) of individuals had a variant(s) associated with a definitive or probable molecular diagnosis (Fig. S1). Of these 141 individuals, 3 had dual definitive and/or probable molecular diagnoses for a total of 144 diagnoses. An additional 12.0% (62/515) of individuals in our cohort carried a variant(s) associated with a provisional diagnosis.

**Fig. 2:**
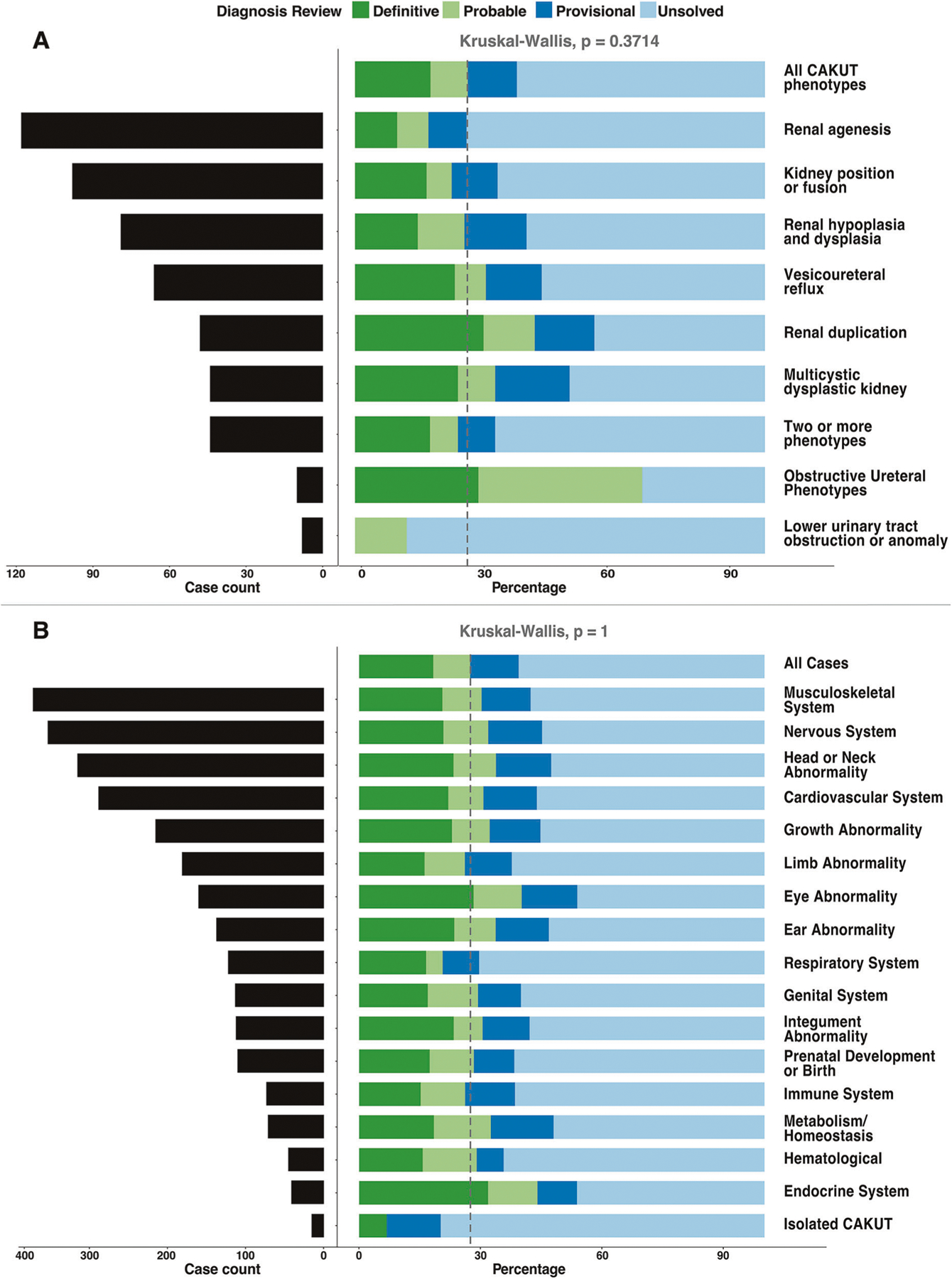
Diagnostic rate of cES for CAKUT. **A** Diagnostic rates of 515 individuals with CAKUT by diagnostic category—definitive, probable, provisional, and unsolved—and by distinct CAKUT subtype. **B** Diagnostic rates of 500 CAKUT+ (syndromic CAKUT) and 15 isolated CAKUT individuals by specific organ system involvement.

We further evaluated the diagnostic rate of cES across age-at-testing groups, CAKUT phenotypes, and additional affected organ systems (CAKUT+) (Fig. 2; Table 1). Most individuals in our cohort had cES during childhood (2–11 years old). We did not observe a statistically significant difference between receiving a definitive or probable diagnosis between age groups (Table 1; *p* value = 0.0680, Chi-square).

In our cohort, 50.5% of individuals were male, 41.4% were female, and 8.2% were unknown/prenatal cases. We did not observe a significant difference in the likelihood of receiving definitive or probable diagnoses compared to receiving provisional or unsolved diagnoses by reported sex (Table 1; *p* value = 0.0572, Chi-Square).

Next, we compared the odds of receiving definitive or probable diagnoses between individual CAKUT phenotypes compared to all other CAKUT cases and did not observe a statistically significant difference by CAKUT phenotype (Fig. 2A, *p* value = 0.3714; Kruskal-Wallis Rank Sum). Finally, there was no statistical difference in the odds of receiving a definitive or probable diagnosis between individuals with isolated CAKUT vs. CAKUT+ (*p* value = 0.0801; Fisher’s exact), or between individuals with CAKUT+ based on other organ systems affected (*p* value = 1; Kruskal-Wallis Rank Sum; Fig. 2B).

### Coverage of commercially available CAKUT gene panels

In our CAKUT cohort, cES revealed 144 definitive or probable diagnoses, including dual molecular diagnoses in three individuals. Based on gene coverage, between 3.5% (5/144) and 14.6% (21/144) of these diagnoses could have been made using one of four commercially available CAKUT gene panels. From these 144 definitive or probable diagnoses, 120 unique genes were involved in our CAKUT cohort. Between 4.2% (5/120) and 8.3% (10/120) of these genes were covered in each of the four commercially available CAKUT gene panels.

### Review of reported disease genes association with CAKUT

**Fig. 3:**
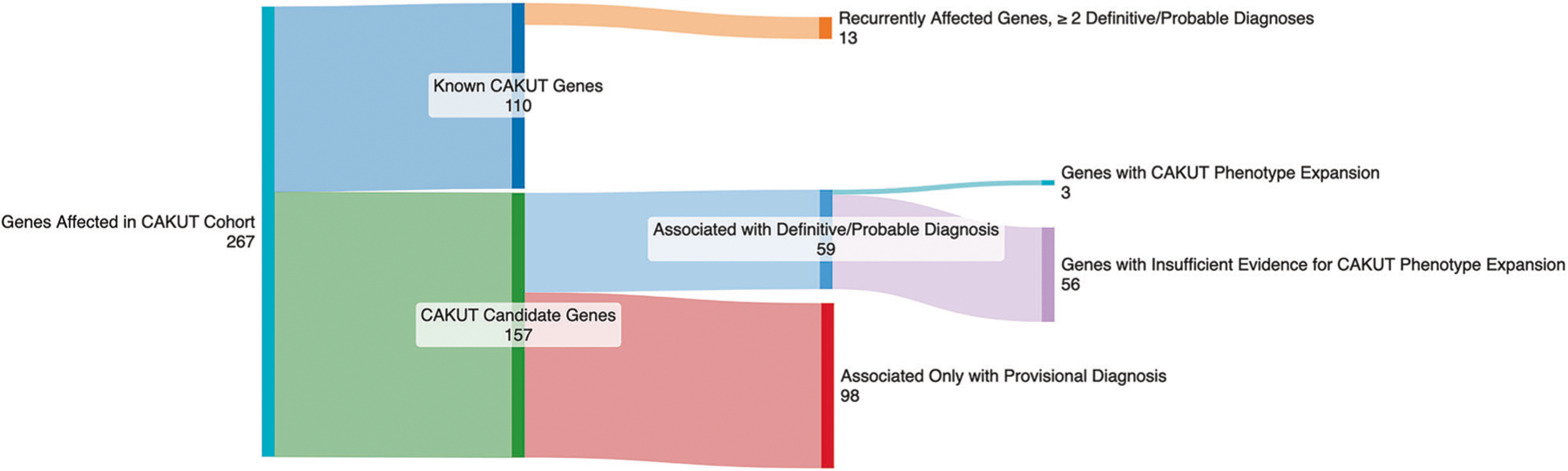
Sanke plot demonstrating the breakdown of 267 genes carrying reported variants from our CAKUT cohort. Of the 267 genes carrying variants reported back to physicians, 110 were genes known to cause CAKUT and 13 of these genes were altered in ≥ 2 individuals in our cohort. Of the remaining 157 CAKUT candidate genes, 98 were only associated with provisional diagnoses in our cohort. Of the 59 associated with a definitive or probable diagnosis, 3 had sufficient evidence to support a phenotypic expansion involving CAKUT, and 56 did not.

We generated and reviewed CAKUT-specific rank annotation scores for the 267 genes listed in the reports of CAKUT individuals (Table S3). A subset of our cohort had variants in genes known to cause CAKUT based on data from commercial panels, OMIM/HPO, or the literature. Among these 110 known CAKUT genes (Fig. 3), *ACTB*, *BRIP1*, *CDK13*, *CDKL5*, *CREBBP*, *CTNNB1*, *EP300*, *FOXP1*, *KAT6B*, *KMT2D*, *PTPN11*, *SON*, and *TFAP2A* were associated with a definitive or probable diagnosis in 2 or more individuals in our cohort. Despite being clearly associated with CAKUT, *BRIP1, CDKL5, CREBBP*, *CTNNB1*, *EP300*, and *FOXP1* do not have CAKUT phenotypes listed in the clinical phenotypes associated with their various disorders in OMIM (December 2024) [32].

The remaining variants reported back to physicians were in 157 genes for which an association with CAKUT had not yet been clearly established. These genes were designated as CAKUT candidate genes.

### CAKUT phenotypic expansions

We evaluated each of the CAKUT candidate genes to determine whether there was sufficient evidence to support a phenotype expansion involving CAKUT based on: 1) whether the variant(s) in the gene led to a definitive/probable diagnosis in one or more patients in our CAKUT cohort, 2) the gene’s CAKUT-specific rank annotation score, 3) published reports in which CAKUT phenotypes were documented in individuals carrying putative pathogenic variants in the gene, 4) the presence of CAKUT phenotypes in transgenic mouse models involving the gene’s homolog, and/or 5) expression of the gene’s mouse homolog in the developing and/or adult mouse urinary tract.

This evaluation yielded four high-priority CAKUT candidate genes—*ADNP, CBL, PHIP*, and *SETD5*. We then performed gene-specific queries of the Baylor Genetics clinical database for each high priority candidate gene to identify additional individuals with likely damaging/pathogenic variants and renal/urinary phenotypes. This review identified 5 individuals (CAKUT_P516-P520; Table S1) not included in our primary cohort whose molecular and clinical data provide additional support for phenotypic expansions involving CAKUT. Other individuals—CAKUT_P349 (*ADNP*), CAKUT_P337 and CAKUT_P303 (*PHIP*), and CAKUT_P306 (*SETD5*) with variants in these genes were in the original cohort. However, these variants were not reported back to the ordering physician and were not taken into consideration when determining cES efficacy.

The homologs of *ADNP, CBL, PHIP*, and *SETD5* are expressed in the mouse urinary tract. In addition, these genes had 1) highly positive (≥ 85%) CAKUT-specific rank annotation scores and ≥ 3 definitive/probable cases reported in our cohort or in the literature, or 2) a positive (> 50%) CAKUT-specific rank annotation score and ≥ 6 definitive/probable cases reported in our cohort or in the literature. The CAKUT-specific rank annotation scores for these genes ranged from 62.2% to 85.6% with a mean score of 76.2% and median score of 78.6% (Fig. 1B).

We then determined the incidence of CAKUT in individuals in the Baylor Genetics database whose cases were solved by variants in each of our high-priority CAKUT candidate genes. This was then compared to a high estimate of CAKUT incidence in the general population; 1:100. As shown in Table 2, the incidence of CAKUT in individuals with *ADNP*-, *SETD5*- and *PHIP*-related disorders in the Baylor Genetics clinical database were significantly higher than the rate of CAKUT in the general population with *P* values of 0.0444, 0.0079, and 0.0002, respectively. We conclude that *ADNP*, *SETD5*, and *PHIP* are CAKUT associated genes (Table 3; Fig. 3, ‘Genes with CAKUT Phenotype Expansion’).

In contrast, the incidence of CAKUT in individuals with *CBL*-related disorder was not statistically different from that of the general population (*P* = 0.0758). Hence, we consider it only a high priority CAKUT candidate gene (Fig. 3: ‘Insufficient Evidence’).

### Other CAKUT candidate genes

We identified 55 other CAKUT candidate genes associated with a definitive or probable diagnoses in our cohort. The CAKUT-specific rank annotation scores for these genes ranged from 12.5% to 96.4% with a mean score of 61.7% and a median score of 67.7% (Fig. 1B). Within this group, *MED13L* and *WNT7A* were of particular interest. We performed gene-specific queries of the Baylor Genetics clinical database for each these genes and identified two individuals in our original cohort with variants in *MED13L*—CAKUT_P252 and CAKUT_P385—which were not reported to their ordering physicians. However, even with these findings, there was insufficient evidence to support a phenotypic expansion involving CAKUT for *MED13L* or *WNT7A*.

The rank annotation scores of training genes were significantly higher compared to those of *ADNP, CBL, PHIP*, and *SETD5*, and the other 55 CAKUT genes with insufficient evidence for phenotypic expansion (*P* values = 0.039 and 0.0001, respectively, Dunn’s test). However, there was no significant difference in the median scores between genes in the strong and insufficient evidence category (*P* value = 0.597).

## DISCUSSION

### Diagnostic efficacy of cES in individuals with CAKUT

cES has emerged as a powerful tool with the potential to provide a precise molecular diagnosis that informs clinical management and improves genetic counseling for affected individuals and their families [33–36]. The evaluation of cES diagnostic efficacy is key in supporting the decision-making process for physicians and setting expectations for patients and other stakeholders [37, 38].

In our CAKUT cohort, cES provided a definitive or probable diagnosis in 27.4% (141/515) of cases, consistent with previous retrospective studies [5, 15, 39]. When provisional diagnoses are included, the diagnostic yield of cES in our cohort increases to 39.4% (203/515). Most provisional cases stem from variants classified as variants of uncertain significance (VUS).

Most individuals in our cohort were referred for cES in childhood. We did not observe a difference in the diagnostic yield between different age groups. Similarly, the diagnostic yield did not differ significantly by specific CAKUT phenotype (Fig. 2A). However, we acknowledge that this may be due, in part, to the paucity of cES referrals for individuals in some categories, particularly obstructive ureteral phenotypes, and lower urinary tract obstruction or anomalies.

Similarly, we did not observe a significant difference between individuals with CAKUT and CAKUT+, likely due to the relatively low number of isolated CAKUT cases (2.9%, 15/515). We also note the possibility that the cES efficacy in isolated CAKUT cases may have been inflated due to selection bias, a failure to report phenotypes that could have resulted in reclassification as CAKUT+, or a positive family history that was not fully documented in their indications for testing.

Among those with CAKUT+, the most common organ system abnormalities were musculoskeletal (372/515, 72.2%). There was no significant difference in diagnostic yield by affected organ system (Fig. 2B).

### Comparisons of cES and CAKUT gene panel efficacy

The ability of cES to interrogate all disease-relevant genes, regardless of their known association with a specific phenotype, leads to an increased ability to make a molecular diagnosis in individuals with CAKUT when compared to a gene panel [40]. Specifically, we found that of the 144 definitive or probable diagnoses in our CAKUT cohort, only 3.5–14.6% (5–21/144) could have been made using one of four commercially available CAKUT gene panels. In an alternative comparison, we found that only 4.2–8.3% (5–10/120) of the genes implicated in a definitive or probable diagnosis in our cohort were covered in one of the four commercially available CAKUT gene panels.

Based on these results, we recommend that cES be considered in individuals with CAKUT+ for whom a molecular diagnosis has not been identified even if gene panel testing was negative. We would expect that the diagnostic yield of clinical genome sequencing (cGS) would exceed that of cES, and that cGS could serve as a first-tier test replacing the use of both chromosome microarray (CMA) and cES [41, 42].

We note that the European Rare Kidney Disease Reference Network (ERKNet) Working Group on Kidney Malformations suggests that genetic testing for CAKUT should begin with a next generation sequencing gene panel or exome sequencing with applied filters that limit the sequence variant results to those found in a prioritized list of seven genes. If negative, the filters can be adjusted to report sequence variants in genes that have been associated with kidney phenotypes in humans or animal models [43]. If no causative gene is identified in these tests, they indicate that trio cES or cGS should be considered for patients for which there is a high suspicion for inherited kidney disease. Our results suggest that the recommendation to consider cES or cGS should be extended to all individuals with CAKUT+ regardless of the clinical suspicion for an inherited kidney disease. This suggestion seems especially appropriate given the high number of individuals with CAKUT+ who were diagnosed based on the identification of a de novo variant (Table S1).

We feel that the potential benefits of identifying a molecular diagnosis through the use of cES or cGS in individuals with CAKUT + outweigh concerns that might be caused by the identification of incidental findings. Specifically, the benefits of identifying a molecular diagnosis can include the development of individualized medical care plans, access to emerging therapies, accurate genetic counseling, improved prognostication, psychological relief, increased levels of social and emotional support through engagement with those who share the molecular diagnosis, and the avoidance of medical waste in the form of unnecessary testing, imaging, and/or medical procedures [44].

### Recognition of CAKUT phenotypes associated with known disease genes

Understanding the full phenotypic range of known disease genes is crucial for delivering appropriate care to individuals for whom a molecular diagnosis has been made [45]. Online Mendelian Inheritance in Man (OMIM) has served as a valuable resource allowing physicians to quickly determine the phenotypes associated with disease genes and their respective genetic disorders [46]. It is unreasonable to expect that OMIM’s clinical synopses will contain descriptions of all possible phenotypes associated with a particular gene/disorder.

Among the genes affected in more than 2 individuals in our cohort, we found 6 genes with clear evidence of an association with CAKUT but whose clinical synopses in OMIM do not contain CAKUT phenotypes. This underscores the importance of performing a detailed investigation to avoid inaccurately concluding that CAKUT phenotypes must be due to genetic factors other than the variants already identified in known disease genes.

### Phenotypic expansions involving CAKUT

A subset of individuals in our cohort had putatively damaging variants in genes that are not currently known to cause CAKUT. Among these CAKUT candidate genes, we identified three for which there is currently sufficient evidence to support a phenotypic expansion involving CAKUT: *ADNP, PHIP*, and *SETD5*.

### ADNP

Helsmoortel-van der Aa syndrome (MIM #615873), an autosomal dominant disorder characterized by impaired intellectual development, autism spectrum disorder, motor delay, hypotonia, visual difficulties, congenital heart disease, gastrointestinal phenotypes, and facial dysmorphisms, is caused by heterozygous variants in activity-dependent neuroprotective homeobox (*ADNP*; MIM #611386) [47]. Three individuals in our original cohort had *ADNP* variants whose phenotypes provide support for a phenotypic expansion involving CAKUT. CAKUT_P132 had a definitive diagnosis of Helsmoortel-van der Aa syndrome and duplicated kidney. CAKUT_P517 and CAKUT_P349 had provisional diagnoses of Helsmoortel-van der Aa syndrome, and polycystic kidney disease and vesicoureteral reflux, respectively. We identified a fourth individual in our clinical database, CAKUT_P516, who also had a definitive diagnosis of Helsmoortel-van der Aa syndrome who had bilateral hydronephrosis.

Asegaonkar et al. described a fetus with a de novo c.2619_2620delCA, p.(D873Efs*7) pathogenic variant in *ADNP* who had a solitary, enlarged kidney [48]. This fetus also carried a c.1868C>T, p.(A623V) VUS (PM2, BP4) in *NRIP1*, a gene implicated in CAKUT (MIM# 618270) [49]. Although this VUS was inherited from the father, who did not have evidence for CAKUT by ultrasound, this case potentially represents an example of a blended phenotype, where both variants may be contributing to the development of CAKUT [50].

Van Dijck et al. summarized the phenotypes of a large cohort of individuals with *ADNP*-associated neurodevelopmental disorder and found that 6 individuals out of 48 had “renal anomalies” which they described as “narrow ureters, bilateral vesicoureteral reflux that was surgically repaired.” The *ADNP* variants present in these individuals were not specified [51].

*ADNP* has a high CAKUT-specific rank annotation score of 85.6%, and *Adnp* is expressed in the mouse urinary system from TS19 (E11) through TS28 (P4-Adult). The incidence of CAKUT among individuals with Helsmoortel-van der Aa syndrome in the Baylor Genetics clinical database is statistically higher than that of the general population (Table 3). This suggests that individuals with Helsmoortel-van der Aa syndrome can present with a variety of CAKUT phenotypes [26].

### PHIP

Pleckstrin homology domain-interacting protein (*PHIP*; MIM# 612870) encodes multiple proteins through alternative splicing, stimulates cell proliferation, has anti-apoptotic activity, and plays a role in the regulation of cell morphology and cytoskeletal organization [52, 53]. Loss-of-function variants in *PHIP* are the cause of Chung-Jansen syndrome (CHUJANS; MIM# 617991) which is characterized by intellectual disability, behavioral problems, obesity, and dysmorphic features [54, 55].

We report five individuals with CAKUT who carry variants in *PHIP*. CAKUT_P74 and CAKUT_P337 have definitive diagnoses of CHUJANS, and CAKUT_P205 has a probable diagnosis of CHUJANS. Their CAKUT phenotypes are vesicoureteral reflux, horseshoe kidney and left ureteropelvic junction obstruction, and left ureteral hypoplasia, respectively. Provisional diagnoses of CHUJANS were made in CAKUT_P303 and CAKUT_P518 who had a malrotated left kidney and hydronephrosis, respectively.

The incidence of CAKUT in individuals with CHUJANS in the Baylor Genetics database is statistically higher than that of the general population (Table 3). Data from the International Mouse Phenotyping Consortium (IMPC) suggest that 50% (2/4) of early adult male mice carrying a single null-allele of *Phip* had enlarged urinary bladders [56]. We also note that *Phip* is expressed in the mouse urinary system from TS19 (E11) through TS28 (P4-Adult) [26], and that *PHIP* has a positive CAKUT-specific rank annotation score of 62.2%. This led us to conclude that *PHIP* was a CAKUT gene.

During our investigation, Fallois et al. independently concluded that *PHIP* is a CAKUT gene based on novel and published cases of CAKUT associated with deleterious variants in *PHIP* [55, 57–60]. They also provide evidence of *PHIP*’s expression in the developing human and mouse kidney. Hence, our findings provide confirmatory evidence that *PHIP* is a CAKUT gene.

### SETD5

Set domain-containing protein 5 (*SETD5*; MIM# 615743) is a methyltransferase that targets histone H3lys36 for trimethylation (H3K36me3) and thereby affects transcription in neural progenitor cells and their derivatives [61]. Intellectual developmental disorder, autosomal dominant 23 (IDDD23; MIM# 615761) is caused by heterozygous loss-of-function variants in *SETD5* [62].

In our original cohort, CAKUT_P111 has a definitive diagnosis of IDDD23 and unilateral renal agenesis. CAKUT_P306 has a provisional diagnosis of IDDD23 and a left multicystic dysplastic kidney, right-sided renal dysplasia, and a history of vesicoureteral reflux and hydronephrosis. In the Baylor Genetics clinical database we also identified CAKUT_P519 and CAKUT_P520 who have definitive diagnoses of IDDD23 and hydronephrosis and kidney abnormalities, respectively. Szczałuba et al. previously described a family segregating a p.(S973*) [NM_001080517.2] pathogenic variant in *SETD5* in which an affected male child had posterior urethral valves [63].

*Setd5* is expressed in the developing mouse urinary system from TS19 (E11) through TS28 (P4-Adult) [26], and the Deciphering the Mechanisms of Developmental Disorders (DMDD) consortium has reported small kidneys in mice that are heterozygous for a null variant in *Setd5* [64]. These data, and SETD5’s high CAKUT-specific rank annotation score of 85.6%, provide strong evidence to suggest that individuals with IDDD23 can present with CAKUT phenotypes.

### CAKUT candidate genes of interest

Although there was insufficient evidence to conclude that deleterious variants in *CBL*, *MED13L* and *WNT7A*, were associated with an increased risk of CAKUT, these genes remain CAKUT candidate genes of interest.

### CBL

Casitas B-lineage lymphoma protooncogene (*CBL*; MIM# 165360) is an E3 ubiquitin-protein ligase that plays a crucial role in cell signal transduction by targeting receptor tyrosine kinases (RTKs) for ubiquitination and degradation [65]. *CBL* variants have been associated with Noonan-like disorder with or without juvenile myelomonocytic leukemia (NSLL, MIM# 613563) also known as CBL syndrome [66]. This disorder is characterized by developmental delay, hematological abnormalities and dysmorphic facial features.

CAKUT_P126 has renal duplication and a definitive diagnosis of NSLL. There are five other published individuals with NSLL and CAKUT phenotypes: one with unilateral renal agenesis and a c.1144A>G, p.(K382E) pathogenic variant [66], one with hydronephrosis and a c.1096–1G>T (previously reported as c.1098–1G>T) pathogenic splice variant [67], one with bilateral hydronephrosis and hydroureters and a c.1096–4_1096–1del pathogenic variant [68], and identical twins with p.(Q358fs) pathogenic variants; one with hypoplastic kidneys and chronic severe vesicoureteral reflux, and the other with renal dysplasia/hypoplasia and severe ureteral reflux [67]. As with most germline *CBL* variants that cause NSLL, these variants are predicted to cause changes in the linker helix region and RING-finger domains of CBL [68].

*CBL*’s CAKUT-specific rank annotation score is 71.9%. Additionally, *Cbl* is expressed in the mouse urinary system from TS19 (E11) through TS28 (P4-Adult). Although we did not demonstrate an increased risk of CAKUT among individuals with NSLL in the Baylor Genetics clinical database, these data suggest that *CBL* should be considered a high-priority CAKUT candidate gene.

### *MED13L* and *WNT7A*

There is currently insufficient evidence to suggest that the other 55 CAKUT candidate genes can cause CAKUT phenotypes. However, their CAKUT-specific rank annotation scores of these genes are higher than would be expected by random chance (Fig. 1B). This suggests that this group of genes may be enriched for true CAKUT genes. Within this group, *MED13L* and *WNT7A* currently have the greatest amount of evidence in support of a phenotypic expansion involving CAKUT.

Impaired intellectual development and distinctive facial features with or without cardiac defects (MIM# 616789), also referred to as Asadollahi-Rauch syndrome (ARS), is caused by heterozygous variants in *MED13L*. CAKUT_P147 has a definitive diagnosis of ARS and vesicoureteral reflux, and CAKUT_P252 and CAKUT_P385 have provisional diagnoses of ARS bilateral renal hypoplasia with end stage renal disease, and crossed fused renal ectopia, respectively. Three individuals with CAKUT phenotypes and de novo *MED13L *variants have been previously described [69–71]. *MED13L* has a positive CAKUT rank annotation score of 73.6%, and *Med13l* is expressed in the mouse urinary system from TS19 (E11) through TS28 (P4-Adult).

Autosomal recessive *WNT7A* variants associated with partial loss of function cause the abnormal limb development characteristic of Fuhrmann syndrome (FS; MIM# 228930), and autosomal recessive variants leading to a complete loss of function cause the more severe Al-Awadi/Raas-Rothschild/Schinzel phocomelia syndrome (AA/RRS; MIM# 276820) [72]. CAKUT_P193 has a provisional diagnosis of FS, renal agenesis, and other phenotypes consistent with this disorder including an absent thumb, an absent radius, and hip dysplasia. Two individuals with phenotypes suggestive of AA/RRS and CAKUT have been previously described [73, 74]. *WNT7A* has a highly positive CAKUT rank annotation score of 92.5%, and *Wnt7a* is expressed in the mouse urinary system from TS21 (E12.5–14) to TS23 (E15) and possibly at TS28 (P4-adult).

### Clinical practice recommendations

Our findings demonstrate the diagnostic efficacy of cES in patients with CAKUT and broaden the phenotypic features associated with genes already known to cause human genetic disorders. We conclude that cES or cGS should be considered in individuals with CAKUT+ for whom a molecular diagnosis has not been identified, and that cES has the potential to identify many diagnoses in individuals with CAKUT+ that would be missed by using a commercial CAKUT panel. Our data also suggest that additional testing aimed at identifying an independent cause of CAKUT may not be warranted in individuals with an *ADNP*-, *PHIP*-, or *SETD5-*related disorder.

## Supplementary Material

Supplemental Subjects and Methods

Supplemental Figure S1

Supplemental Table S1

Supplemental Table S2

Supplemental Table S3

The online version contains supplementary material available at https://doi.org/10.1038/s41431-025-01929-3.

## Figures and Tables

**Table 1.** Overview of 515 CAKUT cases referred to a diagnostic laboratory for cES.

	Definitive	Probable	Provisional	Unsolved	Significance (*P* < 0.05)
Total (%)	94 (18.3)	47 (9.1)	62 (12.0)	312 (60.6)	
Mean age (SD)	5.1 (5.7)	7.7 (10.6)	4.0 (4.9)	5.3 (7.3)	NS
Age Group (%)					NS
Prenatal	5 (11.4)	1 (2.3)	8 (18.2)	30 (68.2)	
Neonatal (<1 mo)	8 (16.3)	1 (2.0)	7 (14.3)	33 (67.3)	
Infant (1 mo–1 yr)	16 (16.0)	8 (8.0)	10 (10.0)	66 (66.0)	
Toddler (1–2 yr)	9 (17.6)	7 (13.7)	8 (15.7)	27 (52.9)	
Childhood (2–11 yr)	40 (22.1)	21 (11.6)	23 (12.7)	97 (53.6)	
Adolescent (11–21 yr)	13 (18.6)	6 (8.6)	6 (8.6)	45 (64.3)	
Adulthood (21+ yr)	2 (10.5)	3 (15.8)	0 (0.0)	14 (73.7)	
Unknown	1 (100.0)	0 (0.0)	0 (0.0)	0 (0.0)	
Sex (%)					NS
Female	39 (18.3)	20 (9.4)	30 (14.1)	124 (58.2)	
Male	51 (19.6)	26 (10)	24 (9.2)	159 (61.2)	
Unknown (Fetal)	4 (9.5)	1 (2.4)	8 (19.0)	29 (69.0)	

SD = Standard deviation, NS = Not significant, mo = months, yr = years.

**Table 2.** Individuals with a molecular diagnosis, with and without CAKUT, in the Baylor Genetics clinical database.

Gene	Phenotypes (MIM#), Inheritance	Individuals with a definitive or probable diagnosis and CAKUT	Individuals with a definitive or probable diagnosis without CAKUT	Percentage with CAKUT	95% Confidence Interval	*P* value^a^
*ADNP*	Helsmoortel-van der Aa syndrome (615873), AD	2	12	14.3%	1.8 to 42.8%	0.0394^b^
*CBL*	Noonan syndrome-like disorder with or without juvenile myelomonocytic leukemia (613563), AD	1	3	25.0%	0.6 to 80.6%	0.0758 (NS)
*PHIP*	Chung-Jansen syndrome (617991), AD	3	2	60.0%	14.7 to 94.7%	0.0002^b^
*SETD5*	Intellectual disability, autosomal dominant 23, (615761), AD	3	11	21.4%	4.7 to 50.8%	0.0056^b^

*NS* Not Significant.

a Each gene is compared to a general population estimate of CAKUT in 1/100 patients.

b Significant = *P* value < 0.05.

**Table 3.** Genes with sufficient evidence to support a phenotypic expansion involving CAKUT.

Gene	CAKUT-Specific Rank Annotation Score	Phenotypes (MIM#), Inheritance	CAKUT Cases (This Study)	CAKUT Cases (Literature)	Mouse Urinary Tract Expression (MGI)	Transgenic Mice with CAKUT Phenotypes? (MGI/DMDD/IMPC)
*ADNP*	85.6%	Helsmoortel-van der Aa syndrome (615873), AD	Total 4: (1D) Renal duplication (1Pv) Polycystic kidney disease (1Pv) Vesicoureteral reflux (1D) Bilateral hydronephrosis	Total 7: (1D) Renal agenesis^48^ (6I) Renal anomalies^51^	Yes	Not Reported
*PHIP*	62.2%	Chung-Jansen syndrome (617991), AD	Total 5: (1D) Vesicoureteral reflux (1D) Horseshoe kidney, ureteropelvic junction obstruction (1Pr) Ureteral hypoplasia (1Pv) Renal malrotation (1Pv) Hydronephrosis	Total 12: (2D-1Pr) Vesicoureteral reflux^57,58^ (1Pr) Renal agenesis^59^ (1Pr) Ureterocele^55^ (1D-2I) Renal hypoplasia^57,60^ (1D) Ureterovesical junction obstruction^57^ (1D-1I) Horseshoe kidney^58^ (1Pr) Atrophic kidney^58^	Yes	Yes: Enlarged bladder
*SETD5*	85.2%	Intellectual disability, autosomal dominant 23, (615761), AD	Total 4: (1D) Renal agenesis (1Pv) Multicystic dysplastic kidney, renal dysplasia, vesicoureteral reflux (1D) Hydronephrosis (1D) Kidney abnormalities	Total 1: (1D) Posterior urethral valves^63^	Yes	Yes: Small kidneys

*AD* Autosomal dominant, *D* Definitive, *DMDD* Deciphering the Mechanisms of Developmental Disorders, *I* Insufficient information to determine diagnostic certainty, *IMPC* International Mouse Phenotyping Consortium, *MGI* Mouse Genome Informatics, *MIM* Oline Mendelian Inheritance in Man, *Pr* Probable, *Pv* Provisional.

## Data Availability

The data generated during this study can be found within the published article and its supplementary files. All variants reported here have been submitted to the ClinVar database (https://www.ncbi.nlm.nih.gov/clinvar/).
