# Supplementary material for: Clinical exome sequencing efficacy and phenotypic expansions involving non-isolated congenital anomalies of kidney and urinary tract (CAKUT+)
Source: Eur J Hum Genet. Author manuscript; Available in PMC 2025 Dec 5. (PMC12669671; doi:10.1038/s41431-025-01929-3)
Supplement: Supplemental Subjects and Methods [file NIHMS2114636-supplement-Supplemental_Subjects_and_Methods.docx]

**SUPPLEMENTAL SUBJECTS AND METHODS**

**Clinical exome sequencing**

All cES data were generated at Baylor Genetics, a Clinical Laboratory Improvement Amendments (CLIA)–certified laboratory. The following quality control metrics for cES were generally achieved: *>*70% of reads are aligned to the target with *>*95% of targeted bases covered at *>*20x reads, *>*85% of targeted bases are covered at *>*40x reads, and mean coverage of targeted bases is *>*100x reads.

**Clinical diagnostic dataset query**

We accessed coded molecular and clinical data stored in the Baylor Genetics Clinical Laboratory database from ~17,000 individuals referred to Baylor Genetics for cES (December 2011 to January 2021) (1). Data available for this study included age at cES referral, sex, phenotypic features provided by the referring clinician (indications for testing), and variant(s) reported back to physicians as potentially contributing to all or a subset of the individuals reported phenotypes. By querying for CAKUT relevant terms including “renal”, “kidney”, “uret”, or “collecting” we identified 946 individuals with a putative diagnosis of CAKUT.

**Cohort identification using Human Phenotype Ontology annotation and natural language processing via Doc2HPO**

We used natural language processing (NLP) to annotate the phenotypic data available for each individual’s test indication using Human Phenotype Ontology (HPO) terms (2, 3). Data pre-processing steps included correction of phenotype spelling errors using Microsoft Word’s spellchecker, and custom-built regular expressions (pattern matching) in R to separate the family history and other information from the clinical descriptions of each proband.

Following pre-processing, we used the National Center for Biomedical Ontology (NCBO) Annotator via Doc2HPO, a published NLP tool, to assign HPO terms from the de-identified phenotype text provided in the testing indication (4). The output was a list of HPO terms (renal and not renal) for each individual. We propagated these annotations with their ancestors in the HPO tree using the R package OntologyX (5). Using this approach allowed us to rapidly determine which cases were associated with CAKUT HPO terms including, but not limited to, renal hypoplasia/aplasia (HP:0008678), renal agenesis (HP:0000104), multicystic kidney dysplasia (HP:0000003), ureteropelvic junction obstruction (HP:0000074), vesicoureteral reflux (HP:0000076), congenital posterior urethral valves (HP:0010957), and renal duplication (HP:0000075).

Of the 946 individuals identified in our original database query, 515 individuals had sufficient clinical information to warrant a diagnosis of CAKUT (n = 15) or CAKUT + (n = 500) (CAKUT_P1-P515). The phenotypes of these individuals were manually reviewed and grouped by CAKUT structural features i.e. renal agenesis, renal duplication, abnormality of the urethra or ureters, renal ectopy/malrotation/fusion, cystic and dysplastic, or multiple of these categories (6, 7). Phenotypes observed in each of these categories included:

*Renal agenesis* – Unilateral and bilateral kidney agenesis.

*Renal duplication* – Duplicated collecting system, partially-duplicated kidney, bifid collecting system.

*Lower Urinary Tract Obstruction or Anomalies* – Congenital posterior urethral valve, urethral atresia, single urogenital sinus.

*Obstructive Ureteral Phenotypes*– Congenital obstructive megaureter, congenital ureterocele, hydroureter, ureteral hypoplasia, ureterocele, ureteropelvic junction obstruction.

*Renal ectopy/malrotation/fusion*– Ectopic kidney, horseshoe kidney, malrotated kidney.

*Multicystic Dysplastic Kidney*– Multicystic kidney dysplasia. Excluded polycystic kidney disease.

*Vesicoureteral reflux*.

Individuals whose indication for testing did not contain sufficient phenotypic information to ascertain that they had a specific CAKUT phenotype (e.g. “kidney abnormalities”), had renal disease without an explicit structural defect (e.g. renal tubular acidosis, kidney stones), or whose only CAKUT phenotype was hydronephrosis, were excluded from our primary cohort and were not considered when calculating cES efficacy.

**Variant classification**

Each reported variant was reclassified as pathogenic, likely pathogenic, variant of unknown significance, likely benign, or benign by an ABMGG-certified laboratory geneticist (January 2024) using the guidelines for sequence variant interpretation by the American College of Medical Genetics and Genomics (ACMG) and the Association for Molecular Pathologists (AMP) (8).

D**iagnostic review**

Each case was reviewed to establish a level of confidence in the molecular diagnosis (definitive, probable, or provisional) based on the assessed pathogenicity of the molecular findings in the genetics report, expected mode of inheritance, and available phenotypic information as previously described (Table S1) (9). Briefly, a diagnosis was labeled as “definitive” if all or a subset of the provided phenotype data were consistent with the disorder, a pathogenic variant(s) was identified, and the individual demonstrated a pattern of disease inheritance consistent with a disorder associated with the reported variant and gene (i.e. monoallelic variants for a dominant disorder, or biallelic variants for recessive).

A diagnosis was labeled as “probable” if all or a subset of the provided phenotype data were consistent with the disorder and any of the following criteria were met: (1) The individual had a germline heterozygous or hemizygous likely pathogenic variant in a gene associated with an autosomal dominant or X-linked disorder, respectively. (2) The individual had a germline pathogenic variant and a likely pathogenic variant or VUS *in trans*, or two likely pathogenic variants *in trans* in a gene associated with an autosomal recessive disorder. (3) The individual was mosaic for a pathogenic or likely pathogenic variant in a gene associated with an autosomal dominant disorder or hemizygous for an X-linked disorder.

A diagnosis was labeled as provisional if all or a subset of the provided phenotype data were consistent with the disorder and if any of the following criteria were met: (1) The individual had only a single, heterozygous pathogenic variant in a gene associated with an autosomal recessive disease. (2) The individual had two VUSs *in trans,* or a likely pathogenic variant and a VUS *in trans* in a gene associated with an autosomal recessive disorder. (3) The individual was female and was heterozygous for a pathogenic, likely pathogenic, or VUS in a gene associated with an X-linked disorder. (4) The individual had a VUS(s) in a gene whose inheritance was consistent with a disorder associated with that gene.

**Calculating diagnostic efficacy for cES**

The diagnostic efficacies were calculated by dividing the number of individuals with a definitive or probable diagnosis by the total number of individuals. When considering whether an individual with multiple genetic findings had a definitive or probable diagnosis, we used the diagnosis with the highest diagnostic certainty.

**Statistics**

To compare the incidences of CAKUT between individuals with specific diagnoses and the general public, we used a two-tailed Fisher’s exact tests performed using a 2×2 contingency

table calculator available through GraphPad QuickCalcs (https://

www.graphpad.com/quickcalcs/contingency1). P-values of < 0.05 were

considered significant. Binomial exact calculations for 95% confidence intervals were calculated using the online Confidence Interval for a Proportion calculator from the UCSF Clinical & Translational Science Institute (<https://sample-size.net/confidence-interval-proportion/>). All other statistical analyses were performed using R/RStudio. Specifically, the functions chisq.test and Kruskal.test were used to compare the cES efficacy across age, sex, and CAKUT phenotype.

**Coverage of commercially available CAKUT gene panels**

To evaluate the coverage of commercially available CAKUT gene panels, we identified genes screened by four commercially available CAKUT gene panels whose descriptive labels were “Congenital Abnormalities of the Kidney and Urinary Tract Panel” (n = 1), “Congenital Anomalies of Kidney and Urinary Tract (CAKUT) Panel” (n = 2), “Congenital Anomalies of the Kidneys and Urinary Tracts (CAKUT) NGS Panel” (n = 1) (December 2024).

To determine the percentage of the definitive and probable diagnoses made by cES in our cohort that would have been identified by each gene panel based on the genes they screen (the number of definitive and probable diagnoses made by cES in genes screened for in the panel/total number of diagnoses made by cES). In an alternative approach, which eliminates the effects of multiple diagnoses made in a specific gene, we then determine the percentage of unique genes involved in a definitive or probable diagnosis made by cES that were screened in each gene panel (the number of definitive and probable diagnosis-related genes identified by cES that are screened in the panel/total number of definitive and probable diagnosis-related genes identified by cES).

**Generating CAKUT-specific rank annotation scores**

We cross-validated our machine learning procedure using a leave-one-out approach (10). Briefly, each gene in the training set was sequentially excluded, and the machine learning algorithm was trained using the remaining genes. Rank annotation scores were then generated for all RefSeq genes, including the excluded training gene. The resulting scores were studentized, and the score of the excluded gene was recorded. We then compared the studentized cross-validated scores of the training set genes to the scores of all other RefSeq genes derived from applying the machine learning model constructed using all training genes. Receiver operating characteristic (ROC) style curves were generated from this comparison in which the effectiveness of the procedure corresponds to the area under the curve (AUC) and above the diagonal line which represents the result that would be generated by chance alone. An omnibus curve produced using fit data from all knowledge sources was positive, indicating that the algorithm could distinguish between the CAKUT-associated genes in the training set and all other RefSeq genes better than random chance.

**Literature searches to identify additional CAKUT cases associated with candidate genes**

Using PubMed, Google Scholar, and Automated Mendelian Literature Evaluation (AMELIE) we searched for reports of individuals with CAKUT who carried putatively damaging variants in candidate genes (11).

**Candidate gene expression and CAKUT phenotypes in transgenic mouse models**

Using data from the Mouse Genome Informatics (MGI) database, we determined whether the mouse homolog of each CAKUT candidate gene was expressed in the urinary tract between Theiler stage (TS)19 and TS28 which corresponds to embryonic day (E)11-E12.5 and postnatal day (P)4-adult (12, 13). Similarly, using MGI and the International Mouse Phenotyping Consortium (IMPC) database, we searched for urinary tract phenotypes in transgenic mice with deleterious variants in the mouse homolog of each gene of interest (12, 14).

**References**

1. Liu P, Meng L, Normand EA, Xia F, Song X, Ghazi A, et al. Reanalysis of Clinical Exome Sequencing Data. N Engl J Med. 2019;380(25):2478-80.

2. Parikh JR, Genetti CA, Aykanat A, Brownstein CA, Schmitz-Abe K, Danowski M, et al. A data-driven architecture using natural language processing to improve phenotyping efficiency and accelerate genetic diagnoses of rare disorders. HGG Adv. 2021;2(3).

3. Robinson PN, Haendel MA. Ontologies, Knowledge Representation, and Machine Learning for Translational Research: Recent Contributions. Yearb Med Inform. 2020;29(1):159-62.

4. Liu C, Peres Kury FS, Li Z, Ta C, Wang K, Weng C. Doc2Hpo: a web application for efficient and accurate HPO concept curation. Nucleic Acids Res. 2019;47(W1):W566-W70.

5. Greene D, Richardson S, Turro E. ontologyX: a suite of R packages for working with ontological data. Bioinformatics. 2017;33(7):1104-6.

6. Hays T, Thompson MV, Bateman DA, Sahni R, Tolia VN, Clark RH, et al. The Prevalence and Clinical Significance of Congenital Anomalies of the Kidney and Urinary Tract in Preterm Infants. JAMA Netw Open. 2022;5(9):e2231626.

7. Murugapoopathy V, Gupta IR. A Primer on Congenital Anomalies of the Kidneys and Urinary Tracts (CAKUT). Clin J Am Soc Nephrol. 2020;15(5):723-31.

8. Richards S, Aziz N, Bale S, Bick D, Das S, Gastier-Foster J, et al. Standards and guidelines for the interpretation of sequence variants: a joint consensus recommendation of the American College of Medical Genetics and Genomics and the Association for Molecular Pathology. Genet Med. 2015;17(5):405-24.

9. Scott TM, Campbell IM, Hernandez-Garcia A, Lalani SR, Liu P, Shaw CA, et al. Clinical exome sequencing data reveal high diagnostic yields for congenital diaphragmatic hernia plus (CDH+) and new phenotypic expansions involving CDH. J Med Genet. 2021;59(3):270-8.

10. Kunisetty B, Martin-Giacalone BA, Zhao X, Luna PN, Brooks BP, Hufnagel RB, et al. High Clinical Exome Sequencing Diagnostic Rates and Novel Phenotypic Expansions for Nonisolated Microphthalmia, Anophthalmia, and Coloboma. Invest Ophthalmol Vis Sci. 2024;65(3):25.

11. Birgmeier J, Haeussler M, Deisseroth CA, Steinberg EH, Jagadeesh KA, Ratner AJ, et al. AMELIE speeds Mendelian diagnosis by matching patient phenotype and genotype to primary literature. Sci Transl Med. 2020;12(544).

12. Blake JA, Bult CJ, Kadin JA, Richardson JE, Eppig JT, Mouse Genome Database G. The Mouse Genome Database (MGD): premier model organism resource for mammalian genomics and genetics. Nucleic acids research. 2011;39(Database issue):D842-8.

13. Theiler K. The House Mouse-Atlas of Embryonic Development: Springer-Verlag New York; 1989.

14. Groza T, Gomez FL, Mashhadi HH, Munoz-Fuentes V, Gunes O, Wilson R, et al. The International Mouse Phenotyping Consortium: comprehensive knockout phenotyping underpinning the study of human disease. Nucleic acids research. 2023;51(D1):D1038-D45.
