# Supplementary material for: Clinical exome sequencing efficacy and phenotypic expansions involving non-isolated congenital anomalies of kidney and urinary tract (CAKUT+)
Source: Eur J Hum Genet. Author manuscript; Available in PMC 2025 Dec 5. (PMC12669671; doi:10.1038/s41431-025-01929-3)
Supplement: Supplemental Figure S1 [file NIHMS2114636-supplement-Supplemental_Figure_S1.docx]

**Supplemental Figure S1. Schematic describing the identification of our original CAKUT cohort and corresponding molecular findings.** Numbers are reflective of patient counts with the highest diagnostic certainty. Three patients with both Definitive and Probable molecular findings, are counted only in the ‘Definitive’ category to avoid duplicate counting. Abbreviation: Pts – patients.
